# Peripheral Arterial Compression as a New Adjunct Technique to Cardiopulmonary Resuscitation

**DOI:** 10.3390/healthcare10112194

**Published:** 2022-11-01

**Authors:** Kristen M Quinn, William A Hardy, Samuel W Seigler, Heather L Holman, Jennie H Kwon, Taufiek Konrad Rajab

**Affiliations:** 1 Department of Surgery, Medical University of South Carolina, Charleston, SC 29425, USA; 2 College of Medicine, Medical University of South Carolina, Charleston, SC 29425, USA; 3 Pediatric Cardiothoracic Surgery, Medical University of South Carolina, Charleston, SC 29425, USA

**Keywords:** cardiopulmonary resuscitation, CPR adjuncts, CPR device, chest compressions

## Abstract

The success of cardiopulmonary resuscitation (CPR) is critically dependent on the maintenance of myocardial and cerebral perfusion; therefore, preferential perfusion of these vital organs over non-vital vascular beds, such as the extremities, is desirable. We propose that compression of the femoral and/or brachial arteries during CPR improves resuscitation outcomes.

## 1. Background

The current standard of care for CPR consists of chest compressions, breaths, and vasoactive medications; however, only 22–40% of patients survive and most suffer neurologic impairment [1]. Survival and neurologic impairment depend critically on (1) the time between cardiac arrest onset and chest compression initiation, and (2) the maintenance of sufficient myocardial and cerebral perfusion [2]. The first factor depends on bystanders to engage in chest compressions upon arrest, which is a more difficult element to manipulate. The second factor, myocardial and cerebral perfusion, can be augmented by innovation in CPR care.

## 2. Hypothesis

We hypothesize that the usage of peripheral arterial compression devices during CPR can improve survival and neurologic outcomes by shunting blood away from the extremities and towards the coronary and cerebral circulations.

## 3. Discussion

### 3.1. Interventions to Optimize Cerebral and Myocardial Perfusion

Cardiac and neurologic outcomes are directly related to coronary perfusion pressure (CPP) and cerebral (brain) perfusion pressure (BPP) during CPR in animals and humans [3,4,5,6]. Optimizing CPP and BPP, and thereby cardiac arrest outcomes, relies on simple principles: lowering venous pressure or increasing arterial pressure. Investigations in the 1980s assessed venous compression with military anti-shock trousers during CPR and did not show an improvement [7]. Vasoactive agents function to increase the arterial pressure and have been a mainstay of CPR; however, they cause non-selective vasoconstriction, affecting vital and non-vital vascular beds equally. Furthermore, epinephrine, the most common vasoactive drug used in CPR, has beta-adrenergic effects that increase myocardial and metabolic demand, which can exacerbate cerebral and cardiac ischemia.

Another approach to increase CPP is to raise the pressure in the aorta by direct manipulation of the arterial tree. Surgeons have been trained for decades to cross-clamp the aorta during cardiac massage to selectively increase perfusion of the coronary arteries and those vascular beds proximal to the cross-clamp. However, during routine closed-chest compression CPR, the aorta is not accessible for external compression. Aortic occlusion with an endovascular balloon during CPR is a strategy that can accomplish the same result as the cross-clamp when the chest is closed and has demonstrated efficacy in both animals and humans [8,9,10,11,12,13,14]. Endovascular balloon deployment, however, necessitates vascular access obtained by skilled personnel with a sterile technique. This is a time-intensive technique and is not always available for immediate use. Further, this procedure procures complication risks to the large vessels including thrombosis, pseudoaneurysm, and hemorrhage. Thus, aortic balloon occlusion is not practical for routine CPR.

The abdominal aortic and junctional tourniquet (AAJT) is a non-invasive alternative to endovascular balloon occlusion of the aorta. This device is applied to the lower abdomen, and the inflatable bladder functions to occlude the femoral arteries at the level of the umbilicus [15]. Animal studies using the AAJT as a CPR adjunct following traumatic and non-traumatic cardiac arrest found that it significantly increases truncal blood pressure [16,17] and improves survival outcomes [16] compared to animals receiving standard CPR. However, a patient with an unknown pregnancy or an abdominal aortic aneurysm may suffer worse outcomes if the AAJT were used during CPR, and its use on obese patients would be hindered by the difficulty of placing it under their trunk. For these reasons, the use of AAJTs during CPR should not be recommended unless the patient’s medical history is available.

### 3.2. Peripheral Arterial Compression Optimizes Cerebral and Myocardial Perfusion

**Figure 1:**
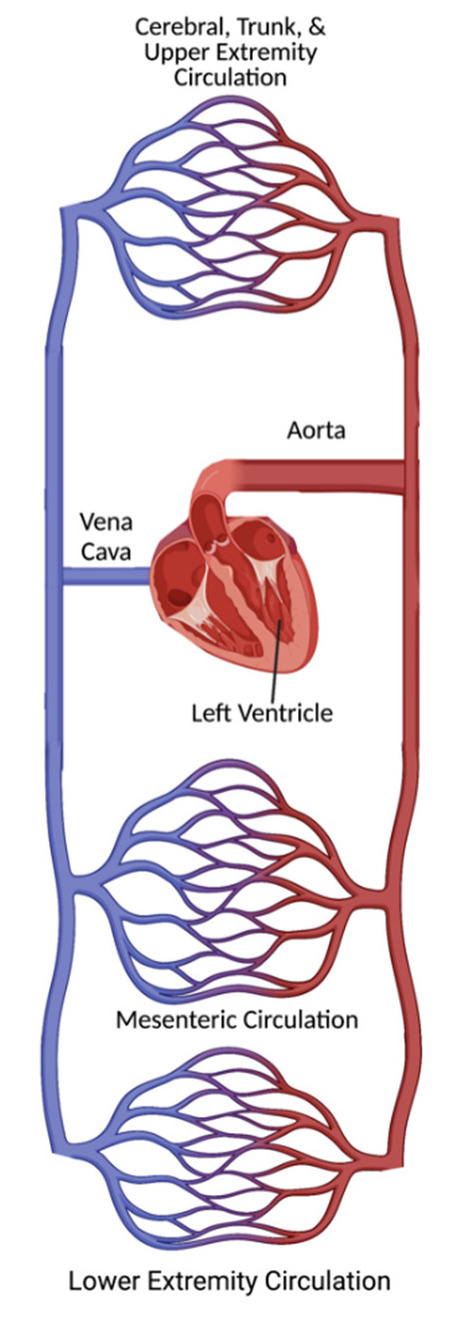
Modeling the human circulatory system as a series of parallel capillary beds.

The systemic circulation has multiple parallel circuits branching from the aorta, permitting a wide variation in regional blood flow at a given cardiac output (CO) (Figure 1). Systemic vascular resistance (SVR) is related to the resistance in each of the parallel circuits (*R*_1_, *R*_2,_ … *R_n_*) according to the equation ${\left(SVR\right)}^{-1}=\left(R_{1}^{-1}+R_{2}^{-1}+\ldots +R_{n}^{-1}\right)$. Substituting this equation into Ohm’s law yields blood pressure (BP) according to the equation 

$BP=CO/\;\left(R_{1}^{-1}+R_{2}^{-1}+\cdots +R_{n}^{-1}\right)$. Removal of parallel circulations theoretically increases BP. This is further supported by the Poiseuille Equation which relates the volumetric flow rate in a long cylindrical tube to the viscosity of the fluid, length of the tube, radius of the tube, and pressure gradient across the tube (Table 1).

**Figure 2:**
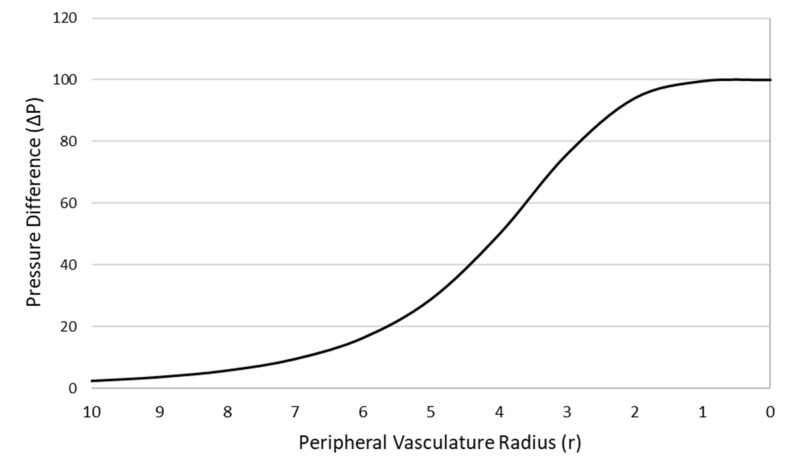
Relationship between the radius of the peripheral vasculature and pressure difference.

By reducing the radius of a single vascular system, the BP would theoretically increase. To understand this relationship in multiple systems, preliminary data was generated (Figure 2). This model shows vasculature with a minimal resistance (large radius) followed by sequentially increasing levels of resistance, resulting in an increase in pressure (BP) across the vascular system. We propose femoral and brachial artery compression as adjuncts to CPR. This intervention shunts blood flow generated by chest compressions away from the extremities and towards the vital organs.

During CPR, only a fraction of the normal 5000 mL/min CO can be generated and made available for the coronary and cerebral circulation. Femoral artery blood flow averages 347 mL/min at rest (Table 2), and although this is likely reduced in the setting of cardiogenic shock, it still represents a large fraction of blood volume [18,19,20,21,22,23,24]. Therefore, occlusion of the extremity vascular beds will shunt the blood volume circulated with compressions to the vital organs (cerebrum and myocardium). Occlusion of the peripheral vasculature also increases SVR, thus raising aortic blood pressures. Peripheral artery compression also prevents resuscitative crystalloids and pharmaceuticals infused during CPR from running off into nonvital vascular beds.

Several large animal studies have demonstrated increased coronary and cerebral perfusion with peripheral vessel occlusion [25,26]. Yang et al. noted that after ventricular fibrillation was induced, pigs with 4-extremity tourniquet-assisted CPR experienced significantly increased coronary blood flow, carotid artery pressure, systolic and diastolic blood pressure, and end-tidal carbon dioxide than the control group without tourniquets providing extremity vessel occlusion [25]. A similar trend was seen in humans when Valli et al. investigated a phenomenon they termed “tourniquet hypertension.” Their study revealed that 27% of patients undergoing limb surgery with a pneumatic tourniquet experienced a 30% increase in systolic and/or diastolic blood pressure [26]. These findings support our hypothesis that peripheral vascular occlusion can increase blood flow to the heart and brain during CPR. 

### 3.3. Limitations of Peripheral Arterial Compression

There are several potential limitations that may prevent the implementation of peripheral arterial compression during CPR. First, occlusion via a tourniquet obstructs both the peripheral arterial and venous structures. The resulting decreased venous return from the extremities would likely cause some reduction in stroke volume and thereby cardiac output. Thus, this technique may potentially be less effective than endovascular arterial occlusion, which obviates this issue by selectively occluding arterial flow. Second, cardiac arrest most commonly occurs in an out-of-hospital setting, and this device might eventually be used by laypersons in a manner similar to an automated external defibrillator (AED). This device would likely need to be applied over clothing, and it may be difficult to deploy around thick clothing. Furthermore, in addition to a rescuer delivering chest compressions and another using an AED, this device may require an additional person’s time and energy to deploy during CPR. Finally, in the emergency department or intensive care unit, select patients may be candidates for extracorporeal cardiopulmonary resuscitation (ECPR). Implementation of a peripheral arterial occlusion device should be deferred in this scenario.

## 4. Conclusions

There is an opportunity to improve cardiac arrest outcomes through increasing blood flow to the heart and the brain during CPR. Peripheral vascular occlusion is a strategy for accomplishing this goal as it shunts CO from the extremities to the conserved vasculature and mechanically increases SVR, which increases perfusion pressures of the vital organs. This maneuver represents a new adjunct to CPR that has the potential to improve the poor outcomes which currently exist, and change the clinical landscape of resuscitative practices.

## Figures and Tables

**Table 1:** Physical science equations adapted to model the circulatory system.

I=100 v=1 L=100 Q=1 $R_{1}=1\;$	** $\Delta P_{leg}=\frac{8vLQ}{\pi r^{4}}$ **	** $\frac{1}{R_{T}}=\frac{1}{R_{1}}+\frac{1}{R_{2}}$ **	** $\Delta P_{Tot}=QR_{T}$ **
	***Poiseuille Equation***	***Resistance in Parallel Circuit***	***Ohm’s Law***
$Radius\;\left(r\right)\;$ ^a^	$\Delta P_{leg}=Pressure\;difference\;of\;leg\;(r^{2})\;$ ^b^	$Total\;Resistance\;\left(R_{T}\right)\;$ ^c^	$Pressure\;Difference\;\left(\Delta P_{Tot}\right)\;$ ^d^
20	0.001	0.001	0.159
10	0.025	0.025	2.483
5	0.407	0.289	28.949
2	15.915	0.941	94.088
0	infinite	1	100

^a^ Radius of leg vasculature; ^b^ Pressure difference of blood in lower extremity in a simple series vascular circuit (downstream pressure minus upstream pressure); ^c^ Total vascular resistance in a parallel vascular circuit; ^d^ Pressure difference of blood in lower extremity in a combined series and parallel circuit.

**Table 2:** Summary of prior studies measuring femoral artery blood flow.

Method	Subjects	Age [Years] (Mean)	Femoral Artery Blood Flow [mL/min] (Mean ± SD)	Reference
Local thermodilution	7 healthy volunteers	N/A	567.5 ± 130.7	Ganz et al., 1964 [18]
Duplex US scanning	51 with no clinical evidence of PAD	44	350 ± 141	Lewis et al., 1986 [19]
Duplex US scanning	24 nondiabetic, nonsmoking subjects without history of cardiac disease or PAD	29	371 ± 132	Field et al., 1989 [20]
Duplex US scanning	80 without clinical evidence of atherosclerosis	40	344 ± 135	Lewis et al., 1990 [21]
Duplex US scanning	20 healthy subjects	28	359 ± 114	Hussain et al., 1996 [22]
Duplex US scanning	40 non-hypertensive nonsmokers with ABI > 1, no history of cardiovascular disease	39.7	284 ± 119	Holland et al., 1998 [23]
MRI	50 without clinical evidence of cardiovascular disease and without medication	55.4	354 ± 120	Klein et al., 2003 [24]

SD, standard deviation; US, ultrasound; PAD, peripheral artery disease; ABI, ankle brachial index; MRI, magnetic resonance imaging; N/A, data not available.
